# Revision of the U. S. Pharmacopoeia

**Published:** 1899-07

**Authors:** 


### Revision of the U. S. Pharmacopoeia.

In another part of this issue of the JOURNAL we publish the call of Dr. H. C. Woods, the president, for a meeting of the convention for the decennial revision of the U. S. Pharmacopœia. We ask the earnest attention of our readers to the same.

It is highly important that the medical and pharmaceutical societies and the medical college organizations that are entitled to representation in this convention should appoint their delegates in time, and we can not too strongly urge them to do so. There is much important work to be done, and it should be done carefully and thoroughly, and not in a perfunctory manner by some half dozen men. These ten-year revisions of the pharmacopœia are made for the purpose of winnowing out those drugs and preparations which in the past have been found worthless or nearly so, and of adding those which have been discovered, or in which new therapeutic value has been demonstrated: in short, to bring the work up to date, and keep it abreast with the progress in therapeutics and pharmacy. There is much in the pharmacopœia that is mere chaff, and it should be thrown out.

*Address before the National Prison Association, December 2, 1897.

But by far the most important question that will come up at the meeting, and one which should be definitely settled is,—not the adoption of a standard of strength and purity of drug preparations in general,—for standardization was adopted at the revision of 1890,—standardization by chemical assay,—but it was applied to three, only, of the toxic and powerful drug plants; viz.: Cinchona, Opium and Nux Vomica. The principle was adopted then, and there can be no reason why it should not be applied to all the dangerous drugs that are susceptible of chemical assay; that is to say,—to Belladonna, Calibar, Gelsemium, Hyoscyamus, Ipecac, Stramonium, Veratrum, Podophyllum, Colchicum, Conium, etc. There are others that are not susceptible of chemical test, and so far, the chemist has not been able to determine with accuracy the amount of the active constituent or the relative medicinal strength, say for instance of Digitalis, Aconite, Cannabis Indica, Ergot and Strophantus, and if a standard of strength of this class is ever possible it will have to be determined by physiological assay; by test on living animals. That however is hardly to be hoped for at present, but will come with time, as will laws in all states and even federal laws which will require a standard of strength and purity of all drugs. Serum therapy has been introduced since the last revision, and it will be,—is an indispensable requisite that there should be a standard of potency of a stated dose and a guarantee of strength and purity of every antitoxin used in practice. What would a physician do, if he had placed in his hands a diphtheria antitoxin serum, for instance, of the strength and reliability of which there should be the same uncertainty as attends the tinctures and extracts of the medical drugs for which no standard has been fixed? He wouldn't dare to use it; yet in a vital crisis he uses ergot of unknown strength. Giving tincture of aconite and other preparations of the kind is the merest kind of guess work. Every physician knows that the strength, medicinally, of parcels of drug plants differs widely; hence, tinctures and extracts made from them, even by the same pharmacist, are of varying strength. It requires no argument to convince the merest tyro in medicine of this fact; it is patent, and the reasons are self-evident; it is not worth while to state them. The writer is cognizant of a case in Austin where an old woman took by mistake a teaspoonful of tincture of aconite root, without any more effect than would have been produced by so much water or diluted alcohol.

The medical profession really have a right to demand of the government, both state and national, protection in this matter.

The doctor carries the responsibility of the lives of the people, and yet many of the most valuable resources of his art are unavailable because of the unreliability of important drugs; he does not get results that he has a right to expect from a given drug. Many physicians have abandoned, as dangerous on account of this unreliability, the use of some important drugs of the pharmacopœa. But, as our experience with legislatures in Texas has demonstrated a uniform indifference on the part of lawmakers in general both to the "demands" of the medical profession and their rights to anything, as well as a surprising disregard of that same public health, of which politicians prate till they have "made their election sure," we abandon the "demand," and the "rights" and the "public health" plea, and advise delegates to the revision convention to try argument with a body smaller in numbers, less heterogeneous, and more intelligent than the average law-making body, and to insist upon the adoption, by the committee, of a uniform standard of strength and purity of all drugs that are amenable to assay. Begin at the fountain head; the laws requiring conformity to the standard will come later.

It would seem, however, that it should require no argument to convince a body, as enlightened as it is supposed this one will be, of the necessity of such action as we have stated; yet it is a fact that the last convention of revisers stopped short at three drugs, out of the long list, in requiring a definite strength of their preparations. It should be insisted that the principle having been recognized in these, standardization should be extended to every drug or preparation of same,—that we have named above. A standard of strength and purity should be *guaranteed* by the pharmacist; and until there are laws to compel conformity to such standard, the practicing physician should withdraw his confidence and patronage from every druggist or manufacturing pharmacist who can not give such guarantee.

